# Structural Insights into the Tetrameric State of Aspartate-β-semialdehyde Dehydrogenases from Fungal Species

**DOI:** 10.1038/srep21067

**Published:** 2016-02-12

**Authors:** Qinqin Li, Zhixia Mu, Rong Zhao, Gopal Dahal, Ronald E. Viola, Tao Liu, Qi Jin, Sheng Cui

**Affiliations:** 1 MOH key Laboratory of Systems Biology of Pathogens, Institute of Pathogen Biology, Chinese Academy of Medical Sciences & Peking Union Medical College, No.9 Dong Dan San Tiao, Beijing 100730; 2 Department of Chemistry and Biochemistry, University of Toledo, Toledo, Ohio 43606, USA

## Abstract

Aspartate-β-semialdehyde dehydrogenase (ASADH) catalyzes the second reaction in the aspartate pathway, a pathway required for the biosynthesis of one fifth of the essential amino acids in plants and microorganisms. Microarray analysis of a fungal pathogen *T. rubrum* responsible for most human dermatophytoses identified the upregulation of ASADH (trASADH) expression when the fungus is exposed to human skin, underscoring its potential as a drug target. Here we report the crystal structure of trASADH, revealing a tetrameric ASADH with a GAPDH-like fold. The tetramerization of trASADH was confirmed by sedimentation and SAXS experiments. Native PAGE demonstrated that this ASADH tetramerization is apparently universal in fungal species, unlike the functional dimer that is observed in all bacterial ASADHs. The helical subdomain in dimeric bacteria ASADH is replaced by the cover loop in archaeal/fungal ASADHs, presenting the determinant for this altered oligomerization. Mutations that disrupt the tetramerization of trASADH also abolish the catalytic activity, suggesting that the tetrameric state is required to produce the active fungal enzyme form. Our findings provide a basis to categorize ASADHs into dimeric and tetrameric enzymes, adopting a different orientation for NADP binding and offer a structural framework for designing drugs that can specifically target the fungal pathogens.

*Trichophyton rubrum* is the most prevalent fungal pathogen for human dermatophytoses, accounting for ~ 70% of the total dermatophyte infections^1^. Recent microarray analysis revealed that the expression of a group of genes were upregulated when *T. rubrum* was exposed to human skin, suggesting their roles as virulence factors and the potential for drug targeting against this fungal organism. Among the upregulated genes EL785855 drew our attention because it encodes for an aspartate-β-semialdehyde dehydrogenase (ASADH)^2^. This enzyme catalyzes the second reaction in the aspartate pathway that is essential in amino acid biosynthesis. ASADH converts β-aspartyl phosphate to aspartate-β-semialdehyde (ASA), which is then either converted to homoserine, a common intermediate in the biosynthesis of threonine, isoleucine, and methionine, or is condensed with pyruvate leading to the production of lysine^3^. The aspartate pathway is the only source for the synthesis of one fifth of the essential amino acids for protein production in plants and microorganisms^4^,^5^. In addition, the aspartate pathway provides the upstream source for cell-wall biosynthesis^6^, the protective dormancy process^7^ and virulence factor production^8^. Therefore, it is no wonder that the *asd* gene belongs to the minimal gene set shown to be indispensable for microorganism survival^9^,^10^. It has been demonstrated that disruption of the *asd* gene will be lethal for many microbial pathogens^11^,^12^,^13^, and ASADH does not have homologs in mammalian cells. Therefore, inhibitors targeting ASADH are considered a promising strategy for the development of novel biocides^3^. In order to assist the drug design against ASADH, high-resolution structural details and full elucidation of the catalytic mechanism are essential. A large collection of crystal structures for ASADHs have been determined to date^3^,^14^,^15^,^16^,^17^,^18^,^19^. Crystallographic data demonstrates that although ASADHs from a variety of organisms exhibit significant sequence diversities (ranging from10 to 95% homology comparing to the prototype *Escherichia coli* ASADH, ecASADH), the overall fold, domain organization and active site structure remain conserved. Microbial ASADHs can be categorized into three branches based on sequence alignment and structural comparison, the Gram-negative branch, Gram-positive branch, and archaeal/fungal branch^3^. The overall structure of the ASADH monomer contains an N-terminal co-enzyme binding domain and a C-terminal dimerization domain consisting of mixed parallel β-strands flanked by α-helices. The central β-strands of two monomers interact with each other to build a homodimer with a local 2-fold symmetry^14^,^18^,^19^. Hinge residues were identified in both the N- and C-terminal subdomains, which facilitate a marked rotational movement of the N-terminal domain towards the C-terminal domain upon NADP binding^17^,^19^. Although the hinge residues are mostly conserved within the ASADH family, NADP induced conformational dynamics have been observed in bacterial ASADHs, but not in the archaeal/fungal branch^18^.

Despite the similar overall fold, deletions and insertions have been found among the different branches of ASADH^14^,^18^. One of the most striking features that differentiate the three branches is the central helical subdomain located on top of the bacterial ASADH homodimer, a region which makes a considerable contribution to the dimer interface^3^. In Gram-negative bacteria such as ecASADH, the helical subdomain is organized into a helical-turn-helical structure that is associated in an anti-parallel orientation with the helical subdomain on the other monomer^19^. The helical subdomain in *Streptococcus pneumonia* (spASADH), a representative of Gram-positive bacteria, is 16 amino acids shorter than in the ecASADH structure, therefore this region only folds into a single helix followed by an unstructured loop, leading to a slightly reduced dimer interfacial area^17^. Strikingly, in the archaeal/fungal branch, there is nearly a 50 residues deletion in this region. The representative structures from mjASADH and caASADH shows a complete absence of the helical subdomain^14^,^18^. Because of this missing helical subdomain, the archaeal/fungal ASADHs are more related to the fold found in an archaeal malonyl-coenzyme A reductase (MCR) and in glyceraldehyde-3-phosphate dehydrogenase (GAPDH) rather than in the bacterial ASADHs. MCR and GAPDH are enzymes that are known to be active as tetramers^20^. Additionally, yeast ASADH is also missing this helical subdomain, was shown to be tetrameric in solution and this tetramer is the catalytically active form^21^. Combining these analyses suggests that ASADHs that are missing the helical subdomain are likely to be tetrameric.

In the current study, we report the first structural insights into the tetrameric ASADH from a fungal species. Our crystallographic data revealed a GAPDH-like folding of trASADH in the crystal. The tetrameric state of trASADH was subsequently confirmed by sedimentation analysis and small-angle x-ray scattering (SAXS) analysis in solution. Further, our structural analyses and native PAGE results with other fungal ASADHs demonstrated that ASADH tetramerization is universal in the archaeal/fungal branch. Our functional analyses also indicate that this tetramerization is essential for the catalytic activity of trASADH, because mutations that cause the dissociation of the trASADH tetramer into a homodimer destabilize the enzyme and led to the abrogation of catalysis. Our structural findings also show that replacement of the helical subdomain by the cover loop region in archaeal/fungal ASADH is the likely determinant for this altered oligomerization. The conformational dynamics of the cover loop coupled to coenzyme binding suggest a different NADP recognition mechanism by tetrameric ASADHs. Our findings offer a novel criterion to classify ASADH into dimeric and tetrameric enzymes, and provide a novel structural framework for biocide design specifically targeting the fungal pathogens.

## Results and Discussion

### Overall structure of trASADH

**Figure 1:**
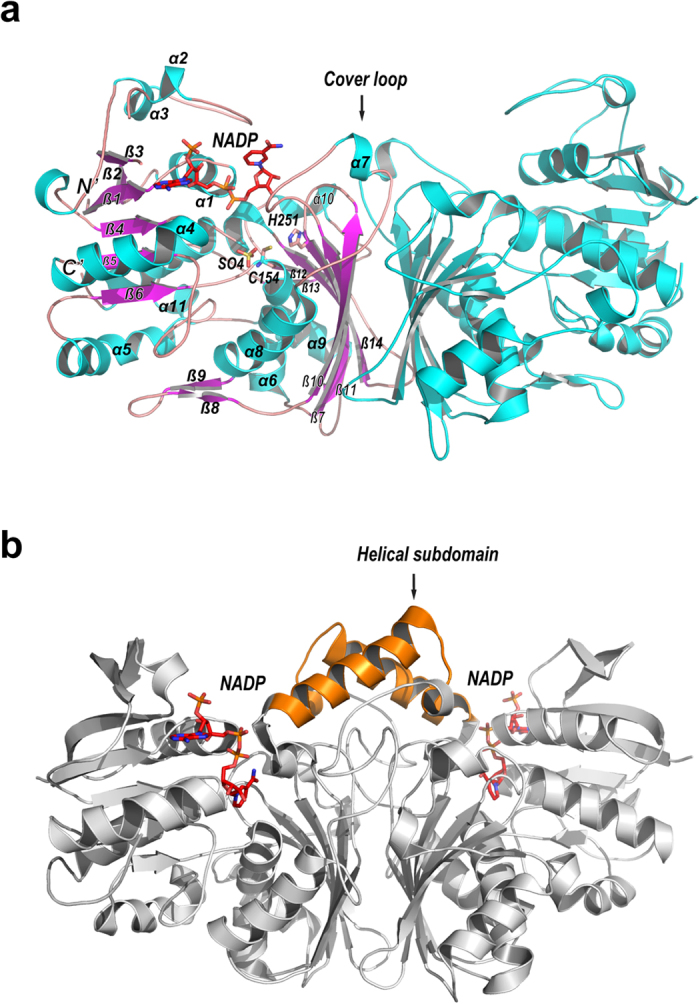
Overall structure of trASADH in dimeric assembly. (**a**) Ribbon model of trASADH (in cyan) in a dimeric assembly. The monomer on the left is colored by secondary structure elements (cyan for α-helix and magenta for β-strand).The secondary structures are labeled. The catalytic important residues, the bound sulfate group at the active site and the coenzyme NADP are shown in stick model and colored by atom type. The “Cover loop” is indicated. (**b**) Ribbon model of spASADH (in gray) homodimer, which adopts the equivalent orientation as trASADH in panel A. The central helical subdomain is labeled and highlighted in orange. The bound NADPs are shown in stick model and colored by atom type.

Recombinant ASADH from *T. rubrum* (denoted: trASADH) was overexpressed in *E. coli* and purified to high purity. Selenomethionine derivatized trASADH was crystallized in its substrate-free state. These crystals diffracted Xray to a resolution limit of 2.6 Å and the crystal structure was subsequently solved using the single-wavelength anomalous diffraction method. The atomic model of trASADH was ultimately refined to excellent stereochemistry quality (Table 1). As anticipated based on sequence similarity, the trASADH monomer adopts a highly similar fold as the other members of the ASADH family (Fig. 1a). Structure comparison demonstrates that the structure of trASADH is closely related to the ASADHs from *Methanococcus jannaschii*, denoted mjASADH and *Candida albicans*, denoted caASADH (Protein Data Bank ID: 1ys4 and 3hsk) belonging to the fungal/archaeal ASADH branch. The r.m.s.d. value is 1.05 Å for 329 aligned Cα atoms between trASADH and mjASADH, and 1.38 Å for 311 aligned Cα atoms between trASADH and caASADH. Each trASADH monomer can be divided into two subdomains, an N-terminal dinucleotide binding domain (α 1–5, α 11 & β 1–6) wrapping in a canonical α/β Rossmann-like fold and a C-terminal dimerization domain (α 6–10 & β 7–14) consisting of a mixed group of parallel β-strands flanked on both sides by α-helices. Two trASADH monomers are related by a local 2-fold symmetry, with their dimerization domains associated to build a dimer similarly to that observed in other ASADH structures. However, compared to bacterial ASADHs, one of the major structural differences in trASADH is that the central helical subdomain stabilizing the bacterial enzyme dimers is completely missing (Fig. 1b). This structural difference is shared by each of the archaeal/fungal ASADHs.

### Crystal contact analysis suggests tetrameric assembly of trASADH

**Figure 2:**
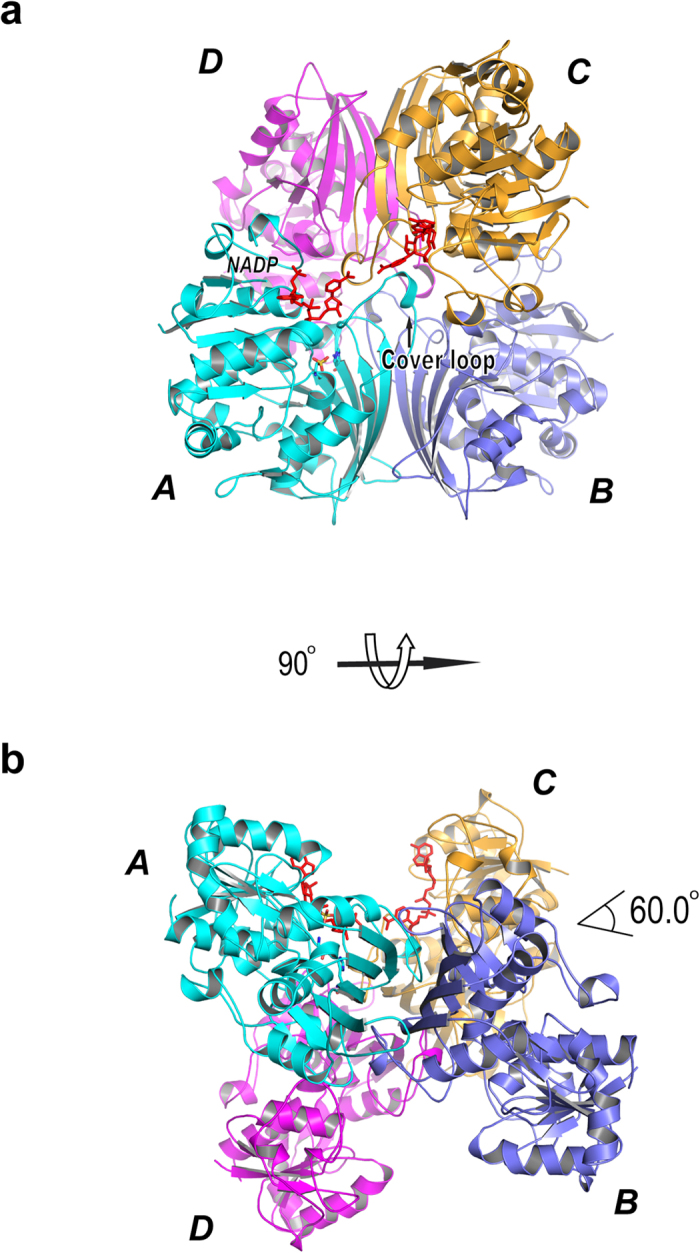
Tetrameric assembly of trASADH. (**a**) Four trASADH monomers are organized into a GAPDH like tetramer, which can best be described as a dimer of dimers. Chain (**A & B**) (colored in cyan and light blue) assemble into a homodimer as shown in Fig. 1a; chain (**C & D**) (colored in orange and magenta) assemble into another homodimer. The bound NADPs are colored in red. (**b**) The tetrameric model of trASADH in panel A is rotated 90° around the x axis, showing that the two dimers are related to each other by an angle of 60.0°.

There are six trASADH monomers (chains A-F) present in the asymmetric unit (ASU) in the current crystal form, whereas only a functional dimer is most often observed in the ASU in other ASADH structures. PDBePISA software was used to analyze the packing interactions^22^. Unexpectedly, this analysis suggests that trASADH organizes into GAPDH^23^-like homotetramers in the crystal (Fig. 2a). There are two tetramer compositions found: chains A-B associated with chains C-D and chains E-F associated with the symmetry-related chains E-F in an adjacent ASU. Both tetramer compositions share the identical organization and similar buried area (12330 Å^2^, 12040 Å^2^), ΔG^int^ (−131 kcal/mol, −127 kcal/mol) and ΔG^diss^(17.1 kcal/mol, 15.1 kcal/mol), respectively. The tetrameric organization of trASADH can best be described as “a dimer-of-dimers”, in which two trASADH dimers are related to each other by an angle of 60° (Fig. 2b).

In a search for structural homologues against the entire public database using software PDBeFOLD^24^, in addition to mjASADH that was found to be mostly closely related to trASADH, another ASADH from *Sulfolobus tokodaii* (stASADH) and an archaeal malonyl-coenzyme A reductase (MCR) also closely resemble the structure of trASADH with the r.m.s.d values of 1.25 Å and 1.32 Å for 348 and 354 Cα atoms, respectively. Interestingly, both of these structural homologues appear to be tetrameric. The stoichiometry of stASADH is indicated to be homotetramer in the crystal (PDB ID: 2ep5); however, there are no further experimental data available to support this conclusion. In the case of MCR (PDB ID: 4dpl), the tetramerization of the enzyme was confirmed both structurally and biochemically^25^^,†^.

**Figure 3:**
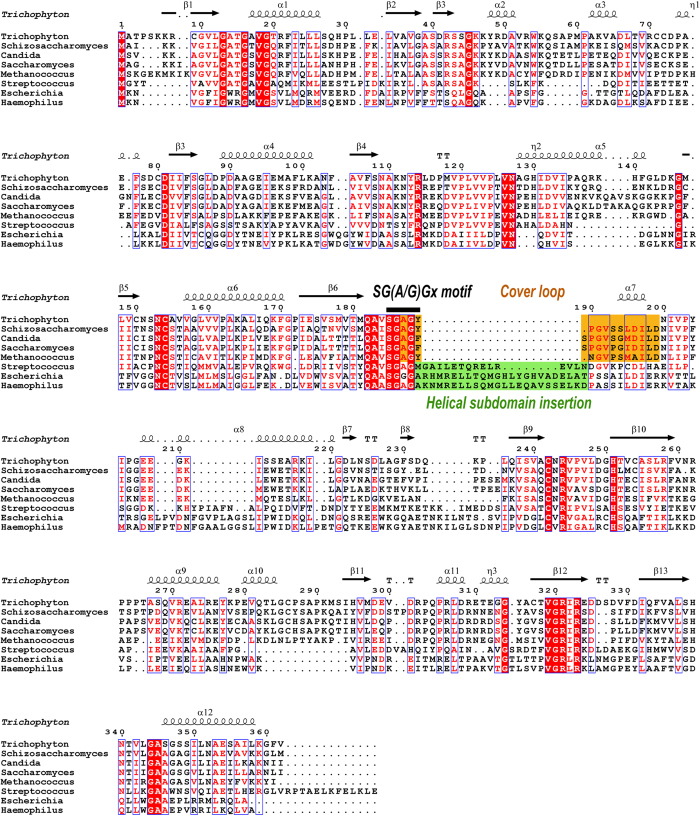
Structure based multiple sequence alignment of various ASADHs. Sequences of ASADHs from various microorganisms are aligned to the sequence of trASADH by the software ClustalW2.The secondary structures of trASADH are superimposed to the sequence on the top of the alignment. The source organisms range from Gram-negative bacteria, Gram-positive bacteria to archaeal/fungal, which are indicated on the left side (*Trichophyton, Schizosaccharomyces, Candida, Saccharomyces, Methanococcus, Streptococcus, Escherichia* and *Haemophilus*). Invariant residues are highlighted with red background; conserved residues are shown in red. The helical subdomains in bacterial ASADHs are highlighted with green background; the cover loops in archaeal/fungal ASADHs are highlighted with orange background.

In the tetrameric model of trASADH suggested by our crystallographic data, a conserved loop from residue 185 to199 replaces the helical subdomain and mediates the dimerization of the two dimers (Fig. 2a). This region is referred to as the “cover loop” in the MCR structure^20^. The dimer-dimer interactions are primarily driven by hydrophobic stacking. For example, the short helix α7 from each dimer are packed against each other via a hydrophobic patch composed of I197 and F198. Given the hydrophobic nature of these surface residues, it is unlikely that the cover loop region can be exposed to solvent, which would be the case in the context of a dimer model. The observed conformation of the cover loop can only be stabilized by this dimer-dimer contact in the context of a tetramer. Sequence alignment and structural comparison show that the cover loop region is highly conserved among the archaeal/fungal ASADHs, but is not present in either Gram-negative or Gram-positive bacterial ASADHs, because these bacterial ASADHs all have the helical subdomain (44–48 aa in length) insertion in this region (Fig. 3). In this regard, archaeal/fungal ASADHs are topologically more related to MCR rather than to their bacterial orthologs. This gives rise to next question: is this tetramerization universal in the archaeal/fungal branch of ASADHs? Crystal packing analysis was performed for the structures of caASADH and mjASADH, which also identified the presence of GAPDH-like homotetramers in each of these structures. The tetrameric assembly of mjASADH containing chains A-B and the symmetry related A-B chains has a buried area of 20320 Å^2^, ΔG^int^ of −119.6 kcal/mol and ΔG^diss^ of 18.6 kcal/mol. A tetrameric assembly of caASADH also contains the same symmetry related dimeric chains and has a buried area of 11940 Å^2^, ΔG^int^ of −55 kcal/mol and ΔG^diss^ of 14.8 kcal/mol. The angles measured between dimer of the dimers were 67.4° and 70.5° for caASADH and mjASADH, respectively (Supplementary Fig. S1). These values are comparable to those observed in the oligomeric assembly in trASADH. In contrast, a similar analysis of the dimeric bacterial structure ecASADH yielded different values: a buried area of only 6740 Å^2^, ΔG^int^ of −51.5 kcal/mol and ΔG^diss^ of 57.5 kcal/mol. Also, the dimeric assembly of spASADH has a buried area of only 5670 Å^2^, ΔG^int^ of −19.9 kcal/mol and ΔG^diss^ of 32.6 kcal/mol. Thus, the tetrameric assemblies in both fungal enzyme structures appear to be more stable than dimer assemblies in these crystal structures, as evidenced by the larger buried areas and lower ΔG^int^ of the tetramers comparing to that of the dimers (Table S1).

Our crystallographic analysis supports the conclusion that archaeal/fungal ASADHs possessing the cover loop but lacking the helical subdomain are tetrameric rather than dimeric, unlike the bacterial enzymes that have been more extensively characterized. The homotetrameric assemblies of trASADH, caASADH and mjASADH exhibit highly similar arrangement of the four subunits, which all resemble the GAPDH tetramer. Interestingly, the structure of ASADH from *Mycobacterium tuberculosis* (Mtb-ASADH) also revealed higher oligomeric assembly than dimer. Vyas *et al.* identified a dodecameric assembly in a crystal packing analysis, however, they finally concluded that only dimer exists in solution^26^. The dimer resembles a typical bacterial ASADH dimer, in which the central helical subdomain insertions stabilize the dimeric assembly. The dodecameric assembly is a spherical complex comprising six ASADH dimers. We further analyzed this assembly and found that the subunit arrangement is fundamentally different from GAPDH like tetramer (Fig. S2). The analysis suggests that a GAPDH like homotetrameric assembly is specific to archaeal/fungal enzymes.

### Fungal and archaeal ASADHs are tetrameric in solution

**Figure 4:**
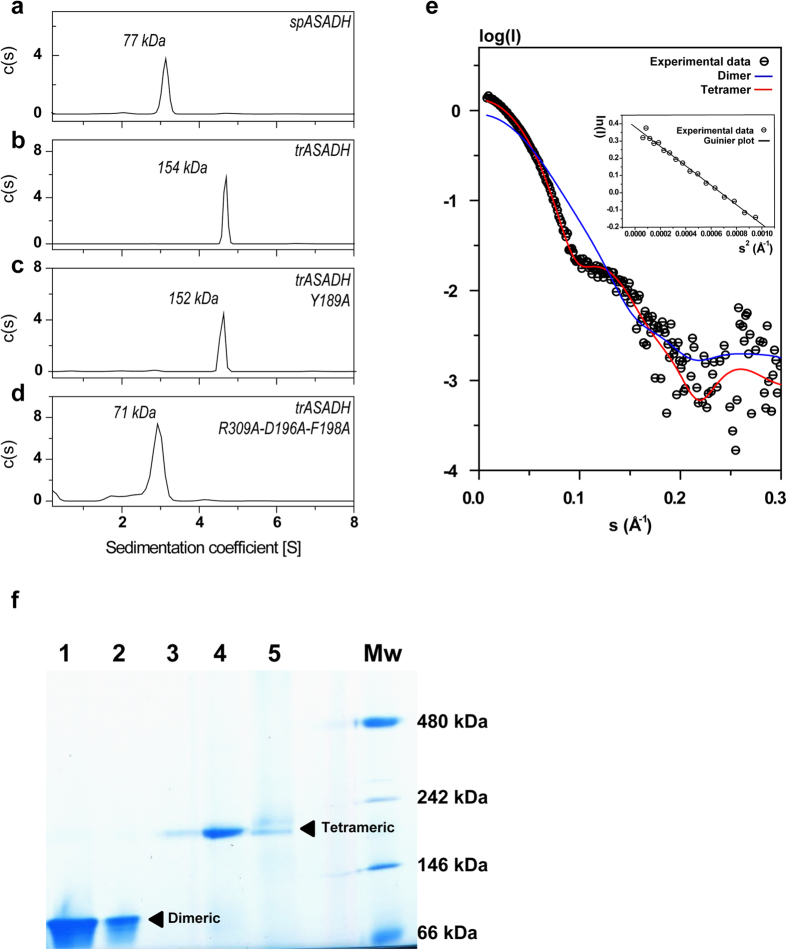
Analytical centrifugation, SAXS and native PAGE analyses reveal tetrameric state of fungal ASADHs. (**a–d**) sedimentation analyses of spASADH, trASADH, trASADH with mutation Y189A and trASADH with mutation R309A-D196A-F198A. The calculated molecular weights are indicated. (**e**) experimental SAXS curve of trASADH (open circle with a dash) is compared with a calculated scattering curve (red) of the tetramer model and the calculated scattering curve (blue) of a dimer model. The upper right insert shows the Guunier plot of the SAXS curve. (**f**) native PAGE (3–12%) analysis shows that the bacteria ASADHs, *S. pneumonia* ASADH (lane 1) and *V. cholerae* ASADH (lane 2) migrated as dimers; whereas the fungal ASADHs, *A. fumigatus* ASADH (lane 3), *C. albicans* ASADH (lane 4) and *C. neoformans* ASADH (lane 5) migrated as tetramers. The lane for the molecular weight standards is labeled.

Whether the trASADH tetramer model suggested by our crystallographic study actually exists in solution demands validation by solution structural analysis. During the final gel filtration purification step, it was observed that the purified trASADH eluted as a single oligomeric species whose size was much bigger than that expected for the theoretical dimer (Supplementary Fig. S3). Next, analytical ultracentrifugation was employed to allow accurate assessment of the solution enzyme molecular weight. Purified trASADH and a bacteria ortholog spASADH were examined at the same concentration, respectively (Fig. 4a,b). The theoretical molecular weights of spASADH (39.2 kDa) and trASADH (38.7 kDa) monomers are similar. SpASADH contains the helical subdomain and is known to be dimeric, thus it served an ideal indicator for dimeric ASADH. The values of S (20,w) were evaluated from the experimental s-value by fitting the data to Sedfit^27^. Sedimentation velocity data of these different ASADHs were analyzed to derive the apparent molecular weight and Stokes radius (Table 2). The apparent molecular weight for trASADH was calculated to be 153.5 kDa, matching the theoretical molecular weight for the tetramer (154.8 kDa). The apparent molecular weight for spASADH was calculated to be 76.7 kDa, matching the theoretical molecular weight for the spASADH dimer (78.4 kDa), which is around half the molecular weight of trASADH. Thus, under similar experimental conditions, the molecular weight assessment supports the tetrameric model for trASADH in solution.

Finally, trASADH was analyzed by small angle X-ray scattering (SAXS) to validate the crystallographic data in a near physiological solution (Fig. 4e). Guinier analyses of the measured SAXS curve for trASADH (2.7 mg/ml) gave a radius of gyration (Rg) of 38.78 Å that is slightly higher than the theoretical Rg value (35.84 Å) calculated from the trASADH tetramer model, but significantly higher than the theoretical Rg (31.29 Å) calculated for the dimer model. This slightly higher Rg value is likely due to some intermolecular attraction among trASADH tetramers in solution. The experimental SAXS curve of trASADH was next compared to theoretical scattering curves calculated either from trASADH tetramer or dimer models by Crysol software. It is clear that the solution structure of trASADH fits the tetramer model with χ = 1.5, in contrast to the significant discrepancy observed in the fitting with the dimer model with χ = 9.4.

To investigate whether tetramerization of ASADH is universal among the members of the archaeal/fungal branch, native PAGE was carried out to analyze additional ASADH orthologs. As shown in Fig. 4f, the fungal ASADHs from *A. fumigatus, C. albicans* and *C. neoformans* (lanes 3–5) each migrated as a tetramer, whereas the Gram-positive bacterial ASADH from *S. pneumonia* and the Gram-negative bacterial ASADH from *V. cholerae* (lanes 1 & 2) migrated as dimers.

Collectively, our results support the conclusion that the ASADHs from the archaeal/fungal branch are each tetrameric in solution, presenting a significant structural difference from the dimeric bacterial ASADHs.

### Homotetramerization is essential for the stability of trASADH

The tetrameric assembly of trASADH is fully supported by our structural and biophysical data. The next question to be answered is whether trASADH is catalytically active as dimers as observed for all other ASADHs reported previously. Based on our crystal structure, the dimer-dimers interaction involves largely hydrophobic contacts and salt bridges in the region of the covering loop. Mutagenesis trials of the dimer-dimer interfacial residues were conducted for the purpose of disrupting these molecular forces, with the goal of disrupting the trASADH tetramer assembly and obtain a dimeric form of trASADH. For each of the single mutations that were produced in this region the outcome of the mutation was either that the tetramer remained unaffected or the mutant led to an intrinsically unstable or completely insoluble protein. This phenomenon is consistent with exposure of the hydrophobic surface of the cover loop at the dimer-dimer interface suggested by our crystal structure; therefore, the tetramerization appears to be of primary importance to the stability of trASADH. Nevertheless, after extensive screening, a mutant bearing the triple mutation R309A, D196A and F198A was found to remain soluble, and also exhibit a dimer size in both size-exclusion chromatography and sedimentation velocity analysis (Fig. 4d). The enzymatic activities of trASADH and the engineered mutants were measured at 25 °C based on the previously established assay^28^. The activity measured for wild-type trASADH reached a *k*_*cat*_ of 16.5 s^−1^. Mutations at the dimer-dimer interface that did not affect homotetramerization of the enzyme also did not have a significant effect on the enzymatic activity. For example, the single mutations at D196A, F198A or R309A did not affect the tetramerization of trASADH, because these mutants all eluted as tetramers in the size-exclusion chromatography (Supplementary Fig. S3). Mutant F198A was subject to ultracentrifugation, which further confirmed the tetrameric assembly in solution (the apparent molecular weight = 143 kDa). The k_cat_ measured for mutants D196A, F198A and R309A are 15.9 s^−1^, 15.7 s^−1^ and 12.9 s^−1^, respectively, indicating a negligible loss of enzymatic activity. By contrast, the catalytic activity of the dimeric triple mutant R309A-D196A-F198A lost nearly all catalytic activity, with the k_cat_ = 0.28 s^−1^, which is less than 2% of that for the wild type enzyme. This observation that the trASADH dimer mutant has negligible enzymatically activity was unexpected. The triple mutations are located at the dimer-dimer interface, whereas the residues in the coenzyme binding pocket and the active site are left unchanged. Circular dichroism spectroscopy analysis of wild type trASADH and spASADH display similar curves, which are distinct from that for the trASADH dimer mutant (Supplementary Fig. S4). This indicates that the dimer mutant is not correctly folded, although it appears to be soluble. Collectively, these results show that tetramerization of trASADH is essential for maintaining the overall folding. We believe that disruption of dimer-dimer interaction may yield the dimeric however misfolded enzyme; therefore trASADH dimer is unlikely active in solution.

### NADP binding

**Figure 5:**
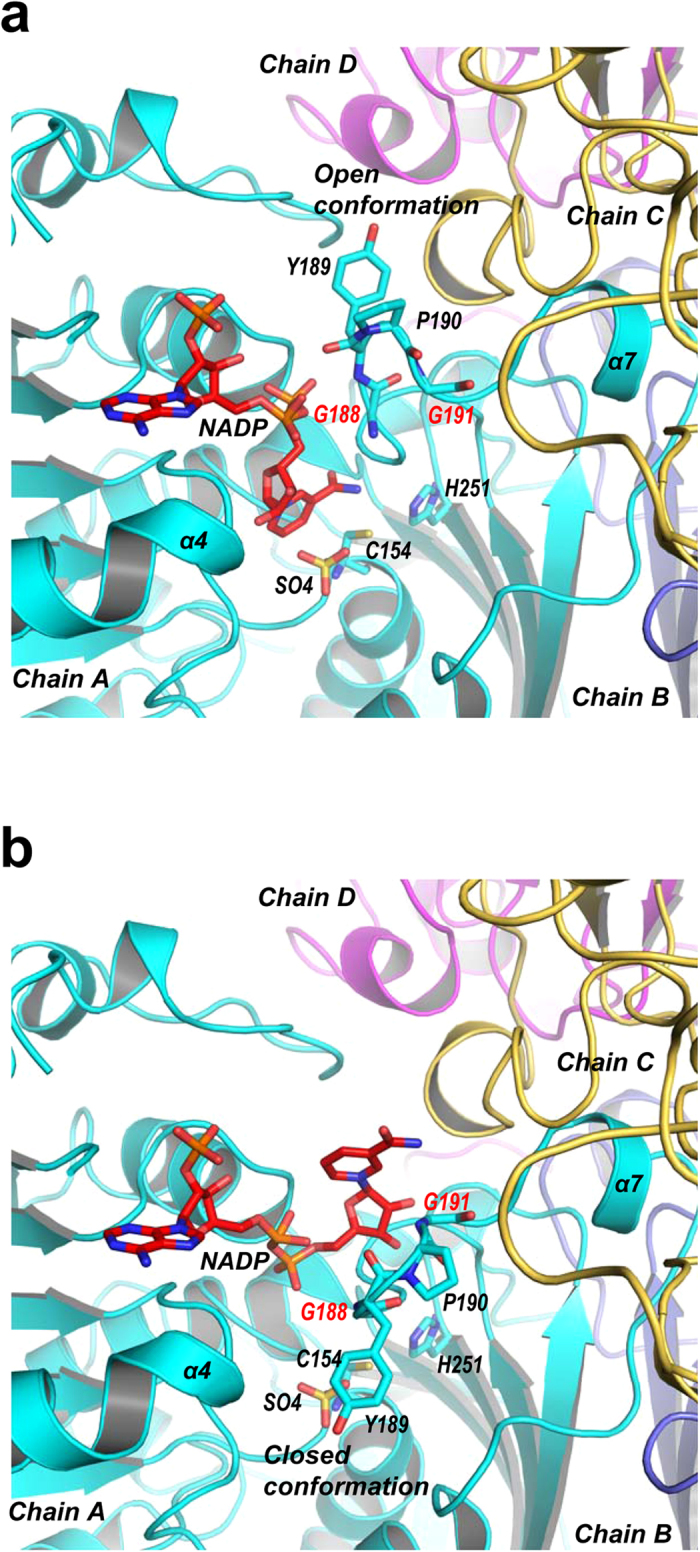
Conformational dynamics of the cover loop upon NADP binding. (**a**) Ribbon model of the coenzyme binding site of trASADH bound by a NADP adopting a canonical S-shaped conformation with the nicotinamide moiety inside the active site. The S-shaped NADP conformation is associated with the “open conformation” of the cover loop. (**b**) Ribbon model of coenzyme binding site bound by a NADP adopting an unusual C-shaped conformation with the nicotinamide moiety positioned outside the active site, representing the catalytic inactive conformation. The C-shaped NADP conformation is associated with the “closed conformation” of the cover loop.

To explore the structural details for coenzyme binding, trASADH was crystallized in the presence of NADP, yielded crystals of trASADH-NADP complex. The complex crystal structure was determined by molecular replacement using apo trASADH as the searching model (Table 1). The electron density for NADPs was only observed in A and C molecules, but not found in four other molecules in ASU, regardless NADP was supplied in an excess during the co-crystallization. In chain A, there was sufficient electron density around the 2’-phospho-ADP moiety of NADP, whereas the electron densities for the rest of the coenzyme become discontinuous, indicating conformational flexibility at this region (Supplementary Fig. S5A,B). This observation also suggests a possibility of low binding affinity of NADP. Therefore we assessed coenzyme binding affinity to trASADH using ITC titration, which gave the following parameters: N/dimer = 1.72 ± 0.308 sites, K_d_ = 76.9 μM, △G = −5.301 ± 1.462 kcal/mol, △H = −5.457 ± 1.462 kcal/mol, T△S = −0.156 kcal/mol, indicating a low binding affinity. Weak binding of the coenzyme to trASADH presents a plausible reason for poor electron density for bound NADP, especially at the nicotinamide moiety. Also, the cover loop adjacent to NADP exhibits poorer electron densities comparing to the same region in the apo structure (Supplementary Fig. S5C). After multiple model building and refinement trials, we found that the electron density map may be best explained by adding the alternative conformations to the bound NADP and to the cover loop spanning residues _188_GYPG_191_. In conformation A (Fig. 5a and Supplementary Fig. S5A), NADP adopts a canonical S-shaped conformation in which the nicotinamide is placed inside the active site with the optimal positon for hydride transfer. In conformation B, NADP adopts a catalytically inactive C-shaped conformation, with the ribose-nicotinamide moiety tilted away and positioned outside the active site (Fig. 5b and Supplementary Fig. S5B). Considering the steric clashes, we speculated that the S- and C-shaped NADP conformations may possibly be associated with different cover loop conformations respectively. The S-shaped NADP conformation is associated with the canonical cover loop conformation as observed in mjASADH and MCR structures reported previously^18^,^20^. Here, the diphosphate of NADP is recognized both by the diphosphate recognition motif _13_GxxGxxG_19_ and by a conserved cover loop region _187_SGAGY_191_, denoted SG(AG/)Gx motif (where x stands for a hydrophobic residue), which combine to fix the position of the diphosphate. In this model the gap between the cover loop and the α4 helix that builds the entrance to the active site has a minimal distance of ~ 10.5 Å; hence, this cover loop conformation is denoted as the “open conformation”. The C-shaped conformation of NADP is associated with a previously unobserved “closed conformation” of the cover loop, in which the _188_GYPG_191_ segment rotates downward around two hinge residues, G188 and G191 (Fig. 5). In this conformation, residue Y189 sitting at the end of the loop is displaced by ~8 Å, thereby narrowing the gap between the cover loop and α4 helix (minimal distance ~ 7.6 Å). The narrowed entrance to the active site could hinder the ribose-nicotinamide moiety entering the active site as illustrated in Fig. 5b. More importantly, when the cover loop adopts this close conformation, the SG(AG/)Gx motif is no longer in the correct conformation to bind to the diphosphate of NADP. The closed cover loop conformation would not be in position to support a catalytically competent NADP conformation, thus presenting an inactive state of the enzyme. In the apo structure, only the close cover loop conformations were observed in all six chains in ASU (Supplementary Fig. S5C).

### Dimeric and tetrameric ASADHs adopt different coenzyme binding strategies

Poor orientation of the nicotinamide moiety of NADP in the ASADH active site, as well as the conformation dynamics of the cover loop, seems to be universal in archaeal/fungal ASADHs^14^,^18^. In the caASADH structure (PDB ID,3hsk), the cover loop region is largely missing in the final model, reflecting the intrinsic flexibility of this region. The bound NADP in this structure also adopts a C-shaped conformation with the nicotinamide moiety positioned outward towards the bulky solvent^14^. Also in mjASADH, there was insufficient electron density for the nicotinamide moiety, regardless of the excess NADP that was present during co-crystallization^18^. By contrast, in bacteria ASADH structures, the occupancy of the NADP is usually high and, while there can be some conformational flexibility in the positioning of the nicotinamide moiety, the electron density for the entire coenzyme is clearly visible^15^,^17^. Our crystallographic findings have revealed the conformational dynamics of the cover loop, which appears to be associated with the conformational changes in the bound NADP. There are two distinct cover loop conformations; the open conformation favors the NADP binding, whereas the close conformation does not. The close-to-open conformational exchange of the cover loop seems to be intrinsic, because the structural transition is mediated by peptide bond rotations at two conserved glycine residues bearing the least dihedral angle constrain. The conformational dynamics of the cover loop could be one of the reasons for poor binding of the nicotinamide moiety, and could be responsible for the lower catalytic activity that is generally observed for the archaeal/fungal ASADHs. The same potential issue does not exist for bacterial ASADHs, because the dynamic cover loop is replaced by a far more rigid helical subdomain that serves to stabilize the conformation in this region.

**Figure 6:**
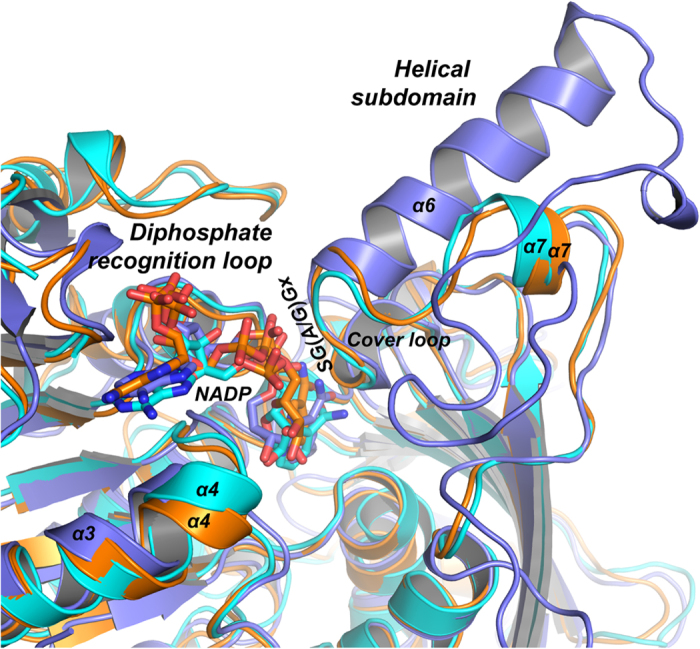
NADP binding by dimeric and tetrameric ASADHs. Portion of the ribbon model of trASADH (cyan) superimposed with mjASADH (orange) and spASADH (light blue). The bound NADPs are shown with stick models and colored by atom type. Regions important for NADP recognition are indicated.

Structural superimposition of a bacterial ASADH (spASADH) with the archaeal/fungal orthologs mjASADH and trASADH in the presence of the catalytically competent NADP was performed to investigate the structural basis underlying dimer-based and tetramer-based coenzyme binding. As shown in Fig. 6, the central helical subdomain (α6) that bridges the top of the β strands at the dimer of dimers interface is replaced by a much shorter cover loop in mjASADH or trASADH. Regardless of this large structural discrepancy between bacterial and archaeal/fungal ASADHs at this site, the folding of the SG(A/G)Gx motif remains superimposable. This is because correct folding of the SG(A/G)Gx loop is required for the direct recognition of the diphosphate of NADP and the subsequent correct positioning of the nicotinamide ring inside the active site. Therefore, stabilization of the open conformation of the SG(A/G)Gx loop appears to be a key component of the catalytic activity. To achieve this, the strategy utilized by bacterial ASADHs is obviously advantageous. The conformation of SG(A/G)Gx loop can be readily stabilized by the secondary structural folding of the immediate downstream helical subdomain. By contrast, both mjASADH and trASADH have a flexible cover loop lacking this stable secondary structure, and the cover loop region also contains multiple exposed hydrophobic patches. Therefore the solution adopted by the fungal ASADHs to stabilizing the cover loop and the SG(A/G)Gx motif is through the interaction with another ASADH dimer to assemble a tetramer. Our analyses offer an explanation for the observation that disruption of the dimer-dimer interface in trASADH often leads to insolubility. In a rare case, the triple R309A-D196A-F198A mutant disrupts the tetramer assembling, but the protein still remained soluble as a dimer. However, this mutant enzyme has lost nearly all of its catalytic activity. This is likely due to the inability of the triple mutant to maintain the proper folding.

## Conclusions

In summary, our findings provide the first structural insight into this tetrameric form of ASADH and offers novel criteria to divide ASADHs into dimeric and tetrameric enzyme families (Supplementary Fig. S6). The tetrameric ASADH is topologically more related to MCR or GAPDH rather than to the bacterial ASADHs. Further, our findings suggest a new possibility for inhibitor design targeting the dimer-dimer interface within the ASADH tetramers. It is widely accepted that protein-protein interactions (PPIs) are promising drug targets with increasing potential^29^,^30^. A growing number of successful examples of PPI inhibitors are emerging, many of which have advanced to clinical trials^31^. Importantly, PPI inhibitors targeting the dimer-dimer interface of ASADH tetramers can be specifically directed to only the fungal enzyme forms. Therefore, this strategy will allow the development anti-fungal drugs with minimal impact on the human bacterial flora.

## Methods

### Constructs and proteins preparation

The cDNA encoding wild-type *T. rubrum* ASADH (362 aa) was amplified from cDNA of *T. rubrum* BMU01672 which was obtained from a patient suffering from tinea unguium^2^. The cDNA fragment was then inserted into plasmid pET-28a for the expression of N-terminal 6 × His-tagged trASADH. Point mutations were introduced using site-directed mutagenesis (QuickChange^TM^). Plasmid encoding wild-type protein or its mutant was transformed into B834(DE3) or Rosetta™(DE3) competent cells (Novagen). The bacteria were grown in LeMASTER medium containing L-selenomethionine at 37 °C to an OD600 of ∼1.0 and cooled to 18 °C before IPTG induction (0.5 mM). The bacterial cells were subsequently incubated at 18 °C overnight. After harvest, the cells were resuspended in lysis buffer containing 50 mM Tris–HCl (pH 8.0), 150 mM NaCl and 10 mM imidazole and disrupted by ultrasonication. The protein purification is composed of three steps. The crude lysate were first loaded onto a Ni-NTA resin (invitrogen) and eluted with 300 mM imidazole. The eluate was further purified using a 5 ml Hitrap Q HP column (GE Healthcare) with a linear gradient of 100–1000 mM NaCl. The polishing step of the purification was size exclusion chromatography using a Superdex 200 HR 10/30 column (GE Healthcare) equilibrated with 20 mM Tris–HCl (pH 8.0), 100 mM NaCl and 2 mM DTT.

### Crystallization and structure determination

Selenomethionine-labeled apo trASADH was concentrated to ∼10 mg ml^−1^ prior to crystallization trials. Optimal crystallization was achieved by mixing 0.8 μl of protein with 0.8 μl of buffer containing 0.2 M Ammonium Sulfate, 0.1 M Sodium citrate (pH5.4) and 15% PEG4000 in a hanging-drop vapor diffusion system at 22 °C. The trASADH-NADP complex crystal was obtained by cocrystallizing the enzyme with 2 mM NADP. The NADP complex crystals were grown in 0.15 M Ammonium Sulfate, 0.1 M Sodium Citrate (pH5.2), 15% PEG4000. Crystals were harvested and subsequently transferred to the reservoir solution containing 15% ethylene glycol, after which the cryoprotected crystal was flash-frozen in liquid nitrogen for data collection. All of the diffraction data were collected at beamline BL17U at SSRF (Shanghai,China) and were processed with the XDS package^32^. The space group identified for apo trASADH was P3121. The programs SHLEX C/D/E were used to locate the heavy atoms and to calculate the initial phases, leading to an interpretable electron density map. The manual model building was carried out by using the program Coot^33^. The structure of the trASADH-NADP complex was solved by molecular replacement (Phaser, CCP4 package) using the structures of Se-Met labeled apo trASADH^34^. The electron density map was improved by manual model building using Coot v0.6^33^. The models were refined with PHENIX^35^. Data collection and the final model statistics are summarized in Table 1.

### Molecular weight determination by size-exclusion chromatography

A Superose 6 PC3.2/30 column (GE Healthcare) was equilibrated with buffer containing 20 mM Tris–HCl (pH 8.0) and 100 mM NaCl. The column was then calibrated using molecular mass standards containing ©-globulin (158 kDa), ovalbumin (45 kDa), myoglobin (17 kDa) and vitamin B12 (1.35 kDa). The purified trASADH (~3 mg/ml) and the other mutants were loaded onto the column running at a flow rate of 0.04 ml/min.

### Analytical ultracentrifugation

Sedimentation velocity was performed with an XL-I analytical ultracentrifuge (Beckman Coulter) equipped with a four-cell An-60 Ti rotor at 4 °C. Reaction buffer containing 20 mM Tris (pH 8.0) and 100 mM NaCl was used as the reference solution. All proteins (OD280 = 0.85) were applied at a speed of 50,000 rpm. Absorbance scans were taken at 280 nm at 0.003 cm intervals in a radial direction. The differential sedimentation coefficients, c(s), frictional coefficients, and molecular weight were calculated by the Sedfit software^27^.

### Small-angle X-ray scattering (SAXS)

SAXS experiments were done using a BioSAXS-1000 instrument (Rigaku). A PILATUS 100 K detector (DECTRIS) was used to record the scattered X-rays at a wavelength of 1.54 Å. The sample-to-detector distance is 500 mm yields the range of momentum transfer (s = 4π sinθ/λ, where 2θ is the angle between the incident and scattered waves) from 0.008 Å-1 to 0.65 Å-1. A human serum albumin (HSA) solution at 5 mg/ml in 50 mM HEPES pH 7.5 was used for calibrating the molecular mass. Scattering data were collected at 3 different protein concentrations (2.7, 5.2 and 11.3 mg/ml) in a buffer containing 20 mM Tris-HCl, pH 8.0, 100 mM NaCl. A standard SAXS data reduction and analysis procedure was done using the program PRIMUS2^36^. CRYSOL^37^ was used to compare the experimental data with the calculated X-ray scattering using the high-resolution models derived from the crystal.

### Kinetic assay

Enzyme assays were performed on a multifunctional microplate reader (SpectraMax M5). Due to the instability of aspartyl phosphate, this reaction is most easily followed in the reverse direction by monitoring the production of NADPH at 340 nm. The assay was performed as follows: 120 mM (pH 8.6) CHES buffer, 200 mM KCl, 0.2~2.4 mM ASA 2.6 mM NADP and 20 mM phosphate were added to a 96-well plate at 25 °C. The enzyme was added to each well to a final volume of 200 μl to initiate the reaction, with a final enzyme concentration of 2 μg ml^−1^ 28.

### Bioinformatic analysis

The sequences of the ASADH proteins obtained from the GenBank database were aligned using the ClustalW software^38^. Sequence alignments were then visualized using ESPript^39^. Phylogenetic trees were reconstructed using the Neighbor-Joining method as implemented in the MEGA5 program^40^. The reliability of the tree was tested via a bootstrap analysis of 1000 replicates.

## Additional Information

**Accession numbers**: The atomic coordinates and structure factors have been deposited in the Protein Data Bank with the accession codes 4ZHS and4ZIC.

**How to cite this article**: Li, Q. *et al.* Structural Insights into the Tetrameric State of Aspartate-β-semialdehyde Dehydrogenases from Fungal Species. *Sci. Rep.* **6**, 21067; doi: 10.1038/srep21067 (2016).

## Supplementary Material

Supplementary Information

## Figures and Tables

**Table 1:** Data collection and refinement statistics.

	ASADH (PDB ID :4ZHS)	ASADH-NADP (PDB ID:4ZIC)
Data collection		
Space group	P 3_1_21	P 3_1_21
Cell dimensions		
a, b, c (Å)	157.50 157.50 188.40	157.338 157.338 187.893
α,β,γ (°)	90.00 90.00 120.00	90.000 90.000 120.000
X ray source	SSRF BEAMLINE BL17U	SSRF BEAMLINE BL17U
Wavelength (Å)	0.979	0.979
Data range (Å)	28.00–2.60	50.00–2.55
Reflections unique*	311564	168846
R_sym_^a^ (last shell)	0.093(0.662)	0.056(0.339)
I / σI (last shell)	7.24(1.56)	19.84(4.37)
Completeness (%) (last shell)	97.2(97.1)	99.7(99.4)
Redundancy (last shell)	1.87(1.87)	3.85(3.83)
Refinement		
Resolution range (Å)	27.62–2.60	46.13–2.55
Reflections (non-anomalous), cut-off, cross validation	159441 (82734) 1.34 8002	87484(87484) 1.35 4385
R_work_^b^/ R_free_^c^ (last shell)	0.214 / 0.255 (0.304/ 0.351)	0.218/0.265 (0.3064/0.4083)
Atoms		
Non-hydrogen atoms	16248	16793
Protein	15823	16113
Ligand	0	144
Solvent	425	536
B-factors average (Å^2^)	55.81	51.27
Protein (Å^2^)	56.04	51.44
Solvent (Å^2^)	47.14	47.44
r.m.s.d		
Bond lengths (Å)	0.007	0.004
Bond angles (°)	1.064	1.062
Validation		
Clashscore, all atoms	13.61	13.36
% Poor rotamers	1.21	1.52
% residues in favored regions, allowed regions, outliers in Ramachandran plot	94.46, 4.72, 0.82	.44 1.12

^*^The unliganded trASADH crystal (PDB ID: 4ZHS) was a selenomethionine derivative, measured at Se edge. F + and F− were considered as separate reflections. Therefore, the unique reflections for 4ZHS is nearly doubled comparing to native crystal of ASADH-NADP complex (PDB ID:4ZIC), in which F + and F− were merged.

*R*_sym_ = ∑_hkl_∑_j_ |I_hkl,j_ - I_hkl_|/∑_hkl_∑_j_I_hkl,j_, where I_hkl_ is the average of symmetry-related observations of a unique reflection *R*_work_ = ∑_hkl_ ||*F*_obs_(hkl)|-|*F*_calc_(hkl)||/∑_hkl_|*F*_obs_(hkl)|. *R*_free_ = the cross-validation *R* factor for 5% of reflections against which the model was not refined.

**Table 2:** Assessment of oligomerization state of ASADHs by sedimentation analysis.

Protein	fitted MW (kDa)	oligomeric state assessment	Ratio to predicted subunit MW	S (20,w)	Rs (nm)
trASADH	153.5	Tetramer	3.8	4.70	7.79
spASADH	76.7	Dimer	1.9	3.14	5.84
trASADH-Y189A	152.5	Tetramer	3.8	4.62	7.87
trASADH R309A-D196A-F198A	77.4	Dimer	1.9	2.85	6.48
trASADH-F198A	143.0	Tetramer	3.5	4.60	7.42

Rs, the Stokes radius was determined experimentally.
