# Supplementary material for: Structural Insights into the Tetrameric State of Aspartate-β-semialdehyde Dehydrogenases from Fungal Species
Source: Sci Rep. 2016 Feb 12;6:21067. doi: 10.1038/srep21067 (PMC4751538; doi:10.1038/srep21067)
Supplement: Supplementary Information [file srep21067-s1.pdf]

Structural Insights into the Tetrameric State of Aspartate-β-semialdehyde Dehydrogenases from Fungal Species

Qinqin Li[1], Zhixia Mu[1], Rong Zhao[1], Gopal Dahal[2], Ronald E.Viola[2], Tao Liu[1], Qi Jin[1]* and Sheng Cui[1]*

[1]MOH key Laboratory of Systems Biology of Pathogens, Institute of Pathogen Biology, Chinese Academy of Medical Sciences & Peking Union Medical College, No.9 Dong Dan San Tiao, Beijing 100730.

[2]Department of Chemistry and Biochemistry, University of Toledo, Toledo, Ohio 43606, USA

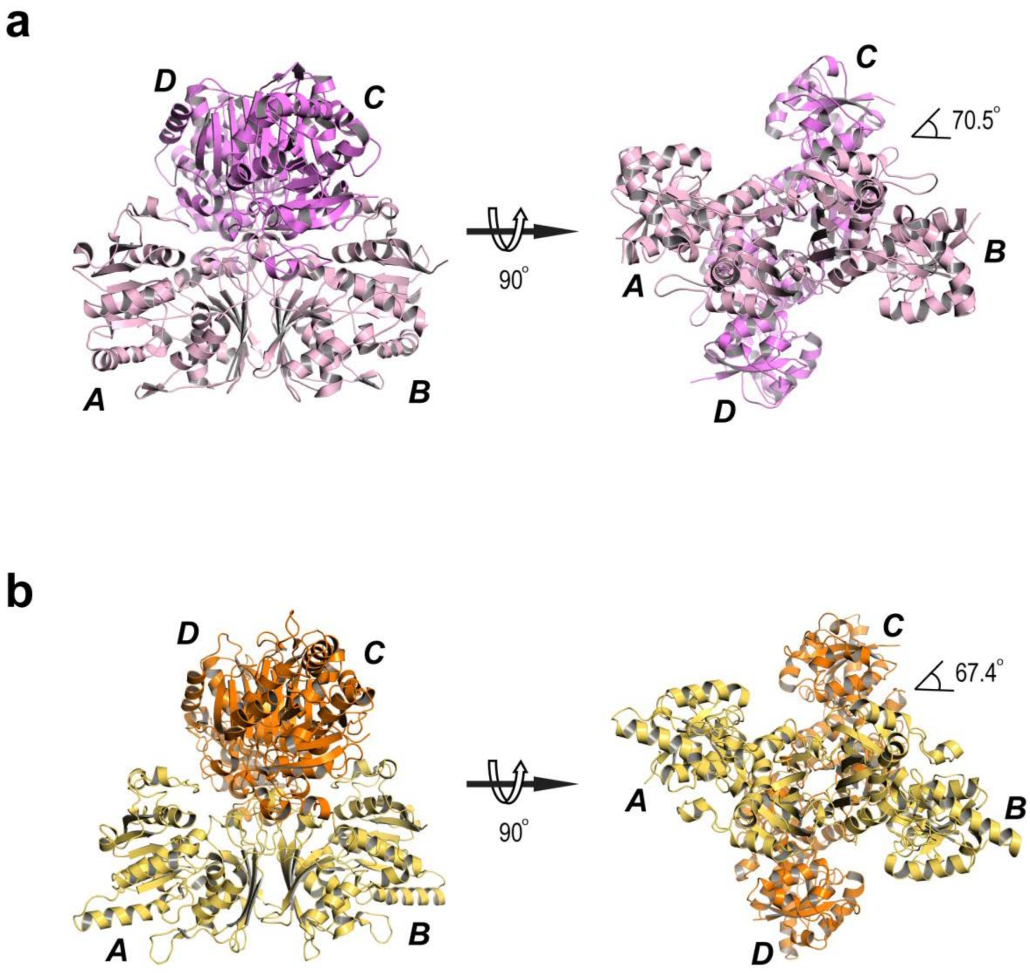

Supplementary Fig. S1, crystal packing analysis reveal tetrameric state of mjASADH and caASADH.

a. Right, ribbon model of the tetrameric assembly of mjASADH identified by software PDBePISA. Left, same model rotates around x axis 90° to show the two dimer of mjASADH are related by 70.5°.
b. Right, ribbon model of the tetrameric assembly of caASADH identified by software PDBePISA. Left, same model rotates around x axis 90° to show the two dimer of csASADH are related by 67.4°.

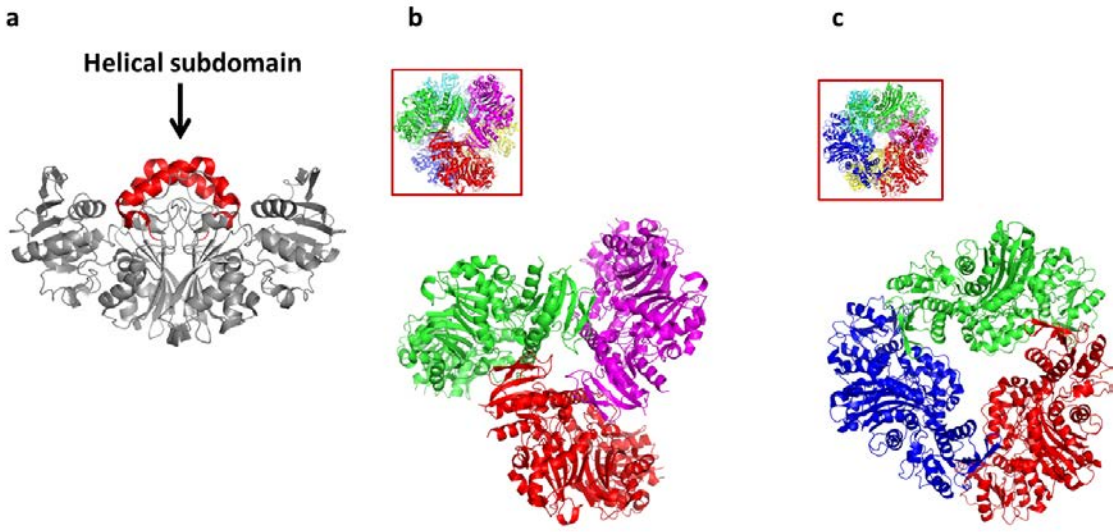

Supplementary Fig. S2, crystal packing analysis of Mtb-ASADH structure reveal a dodecameric assembly that is different from GAPDH like tetrameric assembly.

a. Mtb-ASADH is a typical bacteria ASADH dimer containing the signature helical subdomain insertions (colored in red).

b & c. Mtb-ASADH dodecamer is a spherical complex (red boxes) comprising six ASADH dimers (colored differently). There are two types of trimer of dimers complexes with local 3-fold symmetry. It is clear that none of these assemblies resembles the GAPDH like tetramer assembly as shown in the figure 2 and figure S1.

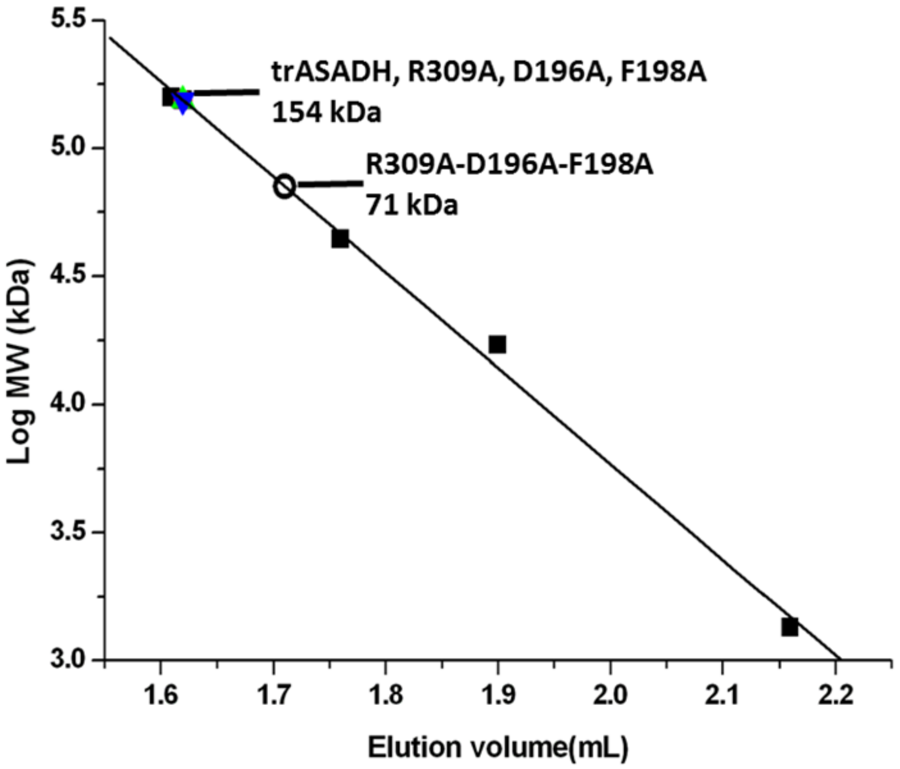

Supplementary Fig. S3, Size exclusion chromatography of various ASADHs.

Semi-logarithmic plot of volume vs. log molecular weight of standard proteins markers: 158kDa, 44 kDa, 17 kDa, and 1.35 kDa, which was used as a molecular standard curve. The molecular weight of trASADH, R309A, D196A, F198A and R309A-D196A-F198A was estimated using the standard curve.

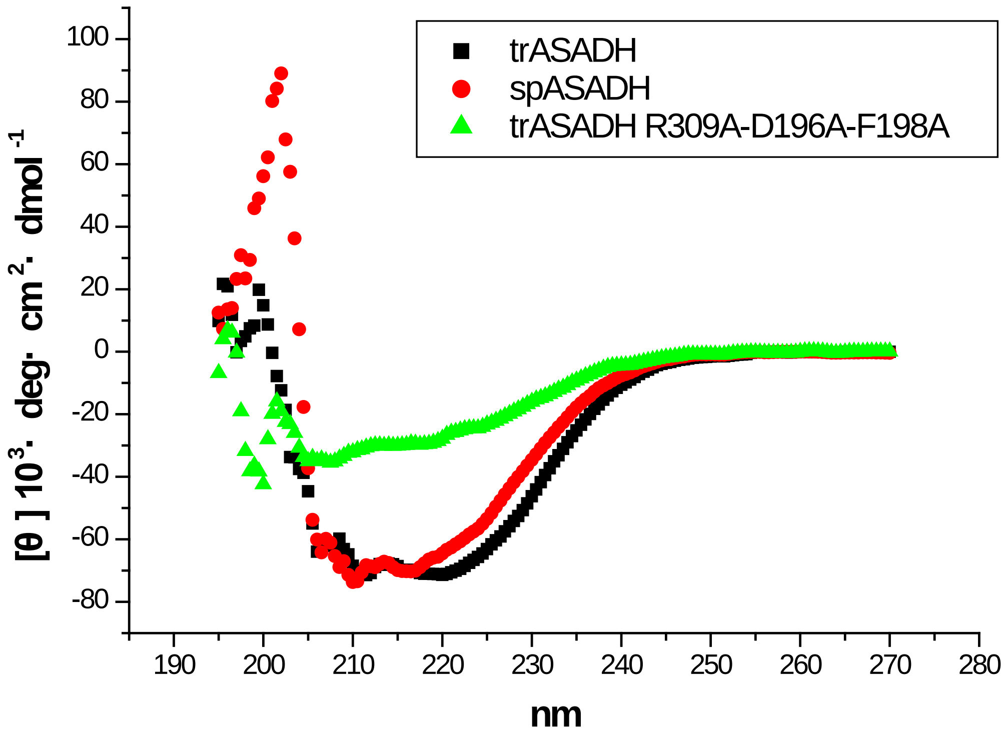

Supplementary Fig. S4, CD spectroscopy of various ASADH and mutant.

CD spectra recorded for trASADH (black squares), spASADH (red circles) and trASADH R309A-D196A-F198A (green triangles).

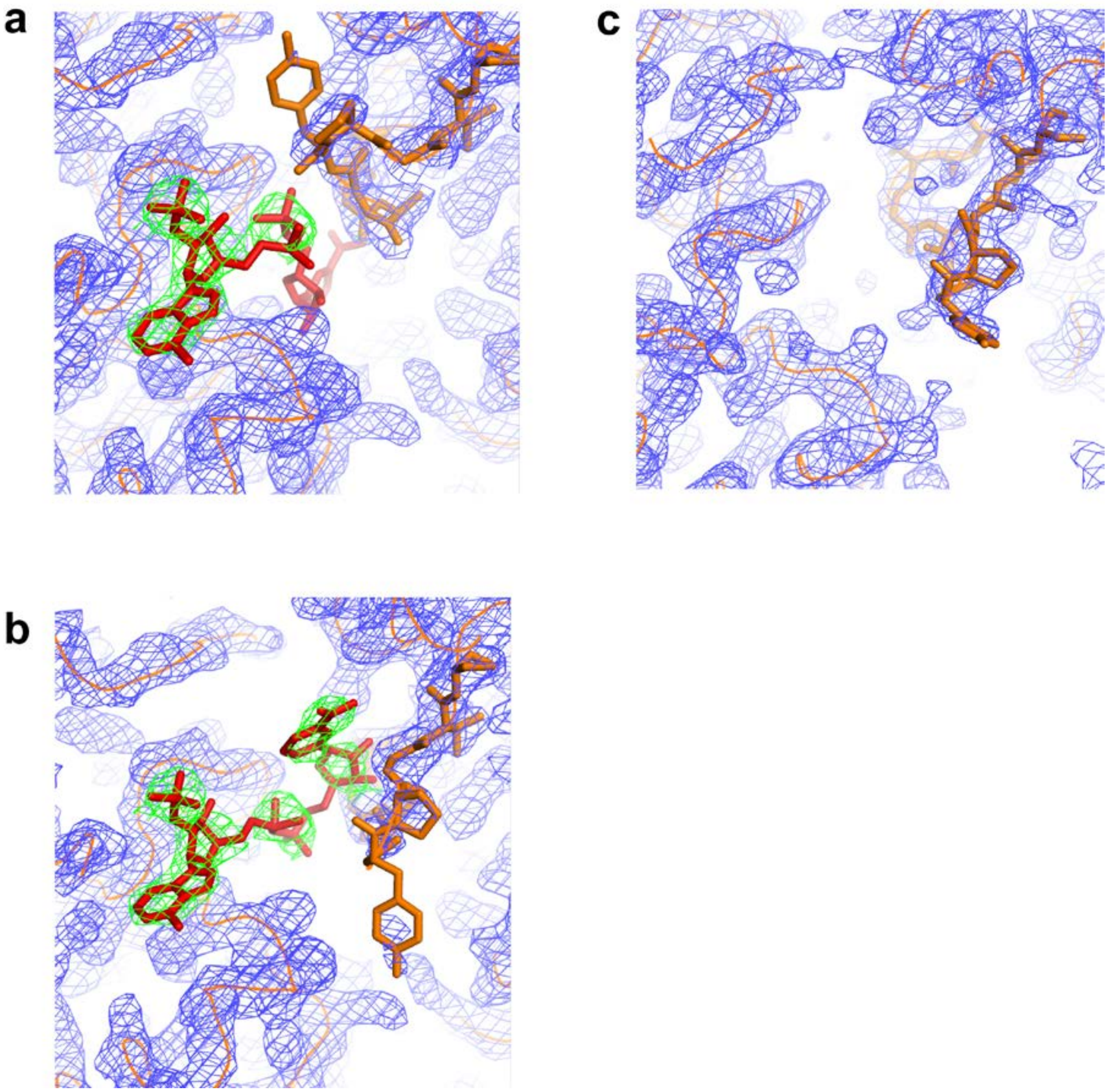

Supplementary Fig. S5, NADP binding to trASADH.

Final models of trASADH and NADP-trASADH complex are superimposed with final 2Fo-Fc map (blue mesh), contour level=1.0. The map for ligand is highlighted with green mesh. NADP is shown in red stick model; trASADH is shown in orange ribbon model. The residues of the cover loop are shown in stick model.

a. the conformation A of NADP (S-shaped) associated with the open conformation of cover loop.

b. the conformation B of NADP (C-shaped) associated with the close conformation of cover loop.

c. The same site as shown in panel A & B in the apo structure of trASADH. The electron density for the cover loop is well defined. The cover loop adopts the close conformation.

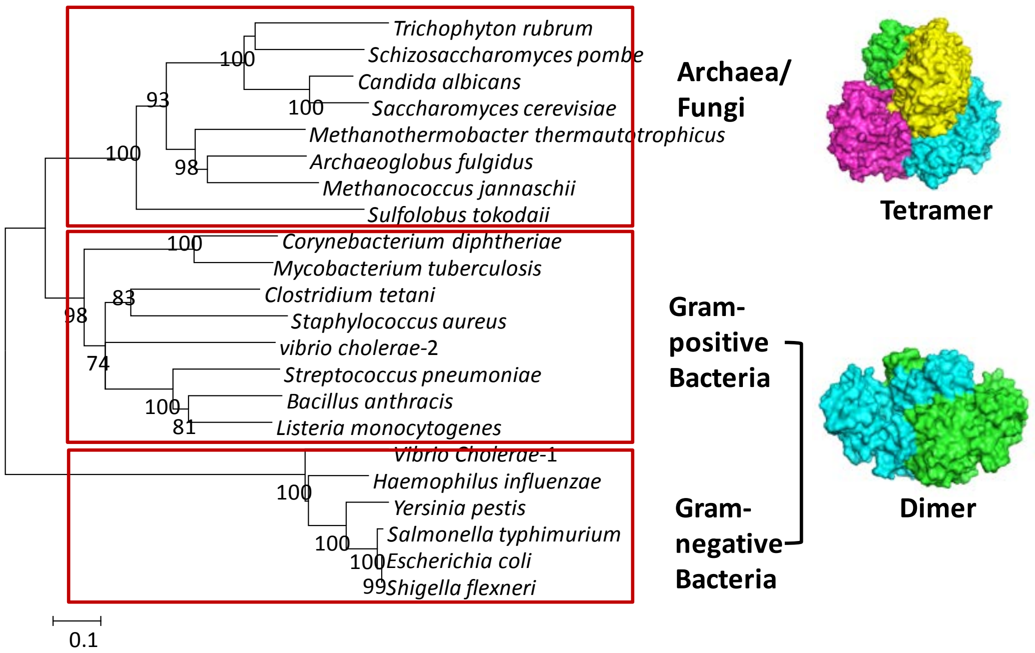

Supplementary Fig. S6, ASADH can be divided into dimeric enzyme and tetrameric enzyme. Phylogenetic tree of ASADH enzymes in microorganisms from a full-length alignment of amino acid sequences. Bootstrap values ⩾ 70% are indicated at the nodes (1000 replicates). Bar, 0.1 residue changes/site.

Supplementary Table S1, PDBePISA analysis of various ASADH oligomers

ASADH	PDB ID	buried area $Å^2$	ΔG^{int} kcal/mol	ΔG^{diss} kcal/mol
Escherichia coli	1BRM[1]	6740	-51.5	57.5
Haemophilus influenzae	1NWC[2]	6810	-49.7	60.8
Streptococcus pneumoniae	2GYY[3]	5670	-19.9	32.6
Mycobacterium tuberculsis	3TZ6[4]	7280	-32.3	34.1
Methanococcus jannaschii	1YS4[5]	20320	-119.6	18.6
Candida albicans	3HSK[6]	11940	-55.0	14.8
Trichophyton rubrum	4ZHS	12340	-131.1	17.1

References

1 Hadfield, A. *et al.* Structure of Aspartate-β-semialdehyde Dehydrogenase from Escherichia coli, a Key Enzyme in the Aspartate Family of Amino Acid Biosynthesis. *Journal of Molecular Biology* **289**, 991-1002, doi:http://dx.doi.org/10.1006/jmbi.1999.2828 (1999).

2 Blanco, J., Moore, R. A. & Viola, R. E. Capture of an intermediate in the catalytic cycle of L-aspartate-beta-semialdehyde dehydrogenase. *Proc Natl Acad Sci U S A* **100**, 12613-12617, doi:10.1073/pnas.1634958100

1634958100 [pii] (2003).
3 Faehnle, C. R., Le Coq, J., Liu, X. & Viola, R. E. Examination of Key Intermediates in the Catalytic Cycle of Aspartate-β-semialdehyde Dehydrogenase from a Gram-positive Infectious Bacteria. *Journal of Biological Chemistry* **281**, 31031-31040, doi:10.1074/jbc.M605926200 (2006).
4 Vyas, R., Tewari, R., Weiss, M. S. & Karthikeyan, S. Structures of ternary complexes of aspartate-semialdehyde dehydrogenase (Rv3708c) from Mycobacterium tuberculosis H37Rv. *Acta Crystallographica Section D* **68**, 671-679, doi:doi:10.1107/S0907444912007330 (2012).
5 Faehnle, C. R., Ohren, J. F. & Viola, R. E. A New Branch in the Family: Structure of Aspartate-β-semialdehyde Dehydrogenase from Methanococcus jannaschii. *Journal of Molecular Biology* **353**, 1055-1068, doi:http://dx.doi.org/10.1016/j.jmb.2005.09.027 (2005).
6 Arachea, B. T., Liu, X., Pavlovsky, A. G. & Viola, R. E. Expansion of the aspartate [beta]-semialdehyde dehydrogenase family: the first structure of a fungal ortholog. *Acta Crystallographica Section D* **66**, 205-212, doi:doi:10.1107/S0907444909052834 (2010).
